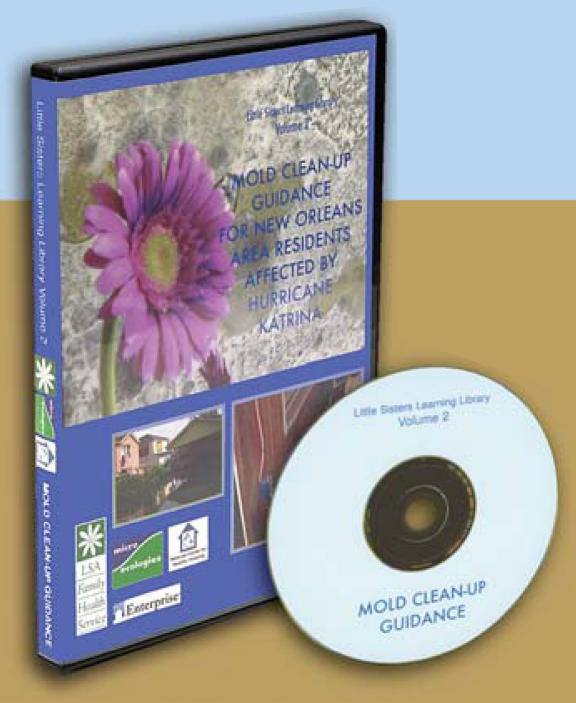# DVD Offers Mold Remediation Tips

**Published:** 2007-06

**Authors:** Tanya Tillett

With homeowners and volunteers—not remediation specialists—performing much of the cleanup and rebuilding along the Gulf Coast following Hurricanes Katrina and Rita, there’s been a greater need for reliable information about avoiding adverse effects of mold exposure. Now a group of several community partners including the Community Outreach and Education Core of the NIEHS Center for Environmental Health in Northern Manhattan (located at Columbia University’s Mailman School of Public Health), the Little Sisters of the Assumption Family Health Service (LSA/FHS) in East Harlem, and the National Center for Healthy Housing has produced a DVD that offers simple, clear instructions on flood-related mold remediation for homeowners, volunteers, and small-scale contractors.

*Mold Clean-Up Guidance for New Orleans Area Residents Affected by Hurricane Katrina* is a 22-minute instructional video that walks viewers through examples of the hazards that can be encountered when cleaning and repairing homes damaged by mold. Narrated by Ray Lopez, the environmental program manager of LSA/FHS, the DVD is divided into eight separate sections that cover a range of topics including recommended personal protective equipment, proper mold cleanup materials and methods, directions for safe handling of personal possessions, steps for safe rebuilding, and general health and safety advisories. Bill Sothern of Microecologies, an indoor environmental investigation firm, helped formulate the cleanup methods described in the video.

Footage for the DVD was collected over several visits to New Orleans in 2005 and 2006 by Lopez and Ginger Chew, an assistant professor of environmental health sciences at the Mailman School of Public Health. The new video builds on guidance offered in a previous LSA/FHS-produced video titled *Learning About Mold*. However, notes Chew, “*Learning About Mold* focuses mainly on mold hazards encountered in high-rise urban city buildings, and this latest DVD directly addresses the mold risks that develop from floodwater exposure such as that which occurred in New Orleans and along the Gulf Coast.”

Lopez explains, “Floodwaters bring their own set of mold problems, especially when combined with [wallboard, with its mold-friendly paper coating], so we felt it was important to go down to New Orleans and produce a video that would be of particular use to the residents there.”

More than 3,700 copies of *Mold Clean-Up Guidance* have been distributed to various relief organizations, and the video is available for viewing or free download at http://www.centerforhealthyhousing.org/html/katrina_video.htm. *Learning About Mold* is out of print but can be viewed online at http://www.littlesistersfamily.org/mold.htm.

## Figures and Tables

**Figure:**